# Ocular drug delivery: a clue from nanotechnology

**DOI:** 10.3389/fphar.2012.00188

**Published:** 2012-10-25

**Authors:** Claudio Bucolo, Filippo Drago, Salvatore Salomone

**Affiliations:** Department of Clinical and Molecular Biomedicine, Section of Pharmacology and Biochemistry, Catania UniversityCatania, Italy

**Figure 1:**
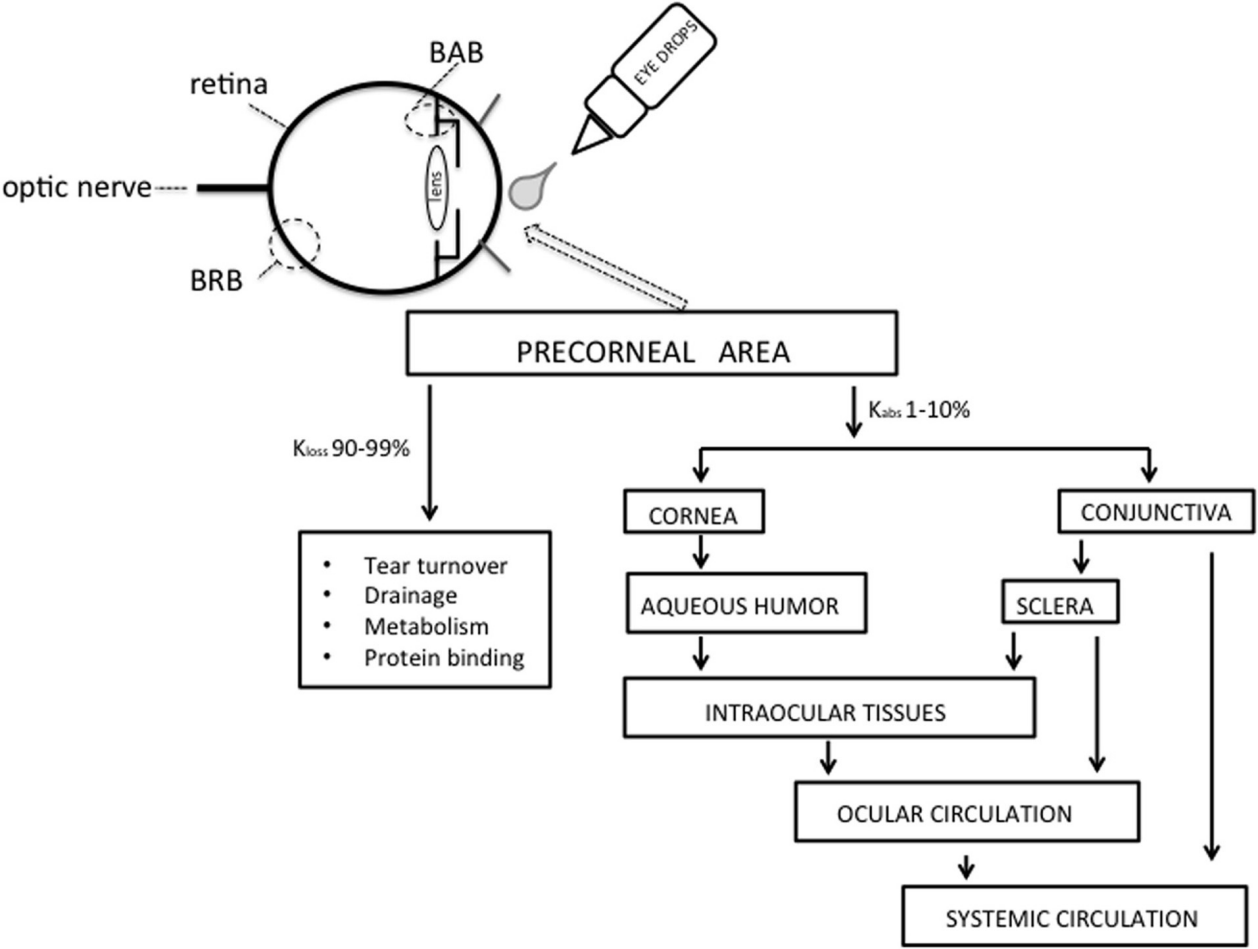
**Model showing the movement of drug into the eye after topical administration.** BAB, blood-aqueous barrier; BRB, blood-retinal barrier.

Topical administration is the most common route of ocular drug delivery. Despite its apparent easy accessibility, the eye is well protected from foreign materials and drugs by a number of very efficient mechanisms such as blinking, induced lacrimation, tear turnover, nasolacrimal drainage, which cause rapid removal of substances from the eye surface and by the cornea, which forms the physical-biological barrier (Figure 1). Consequently, these protective mechanisms and structural obstacle may cause subtherapeutic drug levels at the tissue target, particularly at retinal level. Delivery of drugs to the posterior eye is challenging, and there is an increasing need for managing rapidly progressing for posterior eye diseases, such as diabetic retinopathy, age-related macular degeneration, and optic neuropathy (Bucolo et al., 2012a,b; Musumeci et al., 2012). Currently, the intravitreal route is widely used to deliver therapeutic molecules to the retina. However, frequent administration of drugs *via* this route can lead to retinal detachment, endophthalmitis and increased intraocular pressure. For this reason ophthalmic drug delivery, particularly targeted to posterior segment, is one of the most challenging endeavors facing the ocular pharmacologists. Topical route represents a safer administration, therefore a major challenge to the scientists is to overcome the ocular barriers and reach the tissue target. Systemic route (e.g., oral, parenteral) is also used to reach the eye, though the drug transport across the ocular barriers (blood-aqueous barrier and blood-retinal barrier) is quite difficult.

Under normal condition the human eye can hold about 25–30 μl of an ophthalmic solution; however after a single blink the volume is reduced to 7–10 μl through nasolacrimal drainage which cause the drug to be systemically absorbed across the nasal mucosa or the gastrointestinal tract. A significant systemic loss from topically applied drugs also occurs from conjunctival absorption into the local circulation (Figure 1). Tear turnover, which can also be stimulated by factors such as pH and tonicity of the formulation, remove drug solution from the conjunctival cul-de-sac in a few minutes. The limited permeability of cornea also contributes to the low absorption of ocular drugs. The cornea consists of five distinct layers, with three of them, epithelium, stroma, and endothelium, being the main barriers to absorption. The lipophilic corneal epithelium contains 5–7 layers of cells each connected by tight junctions and represents the rate-limiting barrier for transcorneal diffusion of most hydrophilic drugs; on the contrary the stroma, which is mainly composed of hydrated collagen, exerts a diffusional barrier to highly lipophilic drugs. The endothelium is not a significant barrier to the transcorneal diffusion, in fact its permeability depends on molecular weight rather than characteristic of compound. Usually, 1–10% of the instilled dose is absorbed ocularly and roughly 1% reaches the aqueous humor (Macha et al., 2003). In order to overcome these problems, nanotechnology involving drug-loaded polymers particles has been proposed as ophthalmic drug delivery systems that may control drug release and maintain therapeutic levels over a prolonged period of time. These systems consist of nanoparticles (nanospheres and nanopcapsules). Nanoparticles are polymeric colloidal drug carrier systems with a size ranging from 10 nm to 1 μm, in which drugs are dissolved, entrapped, encapsulated, or to which the drug are adsorbed or attached. Nanospheres are small solid monolithic spheres constituted of a dense solid polymeric network, which develops a large specific area. Nanocapsules are small reservoirs consisting of a central cavity surrounding by a polymeric membrane in which molecules may be dissolved in an oily core or adsorbed to a surface interface. Properly formulated nanoparticles achieve sustained drug release and prolonged therapeutic effect if the formulation is retained in the cul-de-sac after topical administration for a suitable period of time, and the drug is released from the nanoparticles at a proper rate. It is noteworthy that nanosystems are also useful for intravitreal injection delaying the clearance of the drug and reducing the need for repeated injections, thereby lowering the risk of complications.

The success of nanoparticle systems for ocular drug delivery may depend on optimizing lipophilic-hydrophilic properties of the polymer-drug system, optimizing rates of biodegradation, and safety. Polymers used for the preparation of nanoparticles should be mucoadhesive and biocompatible. The choice of polymer plays an important role in the release kinetics of the drug from a nanoparticle system. Ocular bioavailability from a mucoadhesive dosage form will depend on the polymer’s bioadhesion characteristics, which are affected by its swelling properties, hydration time, molecular weight, and degree of crosslinking. The binding of drug depends on the physicochemical properties of the molecule as well as of the nanoparticle polymer, and also on the manufacturing process for these nanoparticle systems. The polymers used in ophthalmic drug formulations are poly(alkyl cyanoacrylates) (PACA) (Das et al., 1995), poly(caprolactone) (PCL) (Losa et al., 1993), poly(lactic acid) (PLA) (Giannavola et al., 2003), poly(lactic-co-glycolic acid) (PLGA) (Vega et al., 2008), chitosan (CS; linear polysaccharide of randomly distributed β-(1–4)-linked D-glucosamine and N-acetyl-D-glucosamine) (Diebold et al., 2007), Eudragit RL100 and Eudragit RS100 [poly(ethylacrylate, methyl-methacrylate, and chlorotrimethyl-ammonioethyl methacrylate) copolymer] (Bucolo et al., 2002, 2004; Pignatello et al., 2002a,b), poly(acrylic acid) (PAA) and hyaluronic acid (Sandri et al., 2006), and modified polystyrene (Jani et al., 2007). In several cases albumin (Irache et al., 2005) gelatin (Vandervoort and Ludwig, 2004) or lipid matrix (Attama et al., 2008) are also used.

In general, the nanotechnology applied to ocular drug delivery systems brought to undoubted advantages such as, among of others, higher solubility, higher area available for dissolution, and higher dissolution rate. Other advantages from nanosystems are higher corneal penetration and lower number of intraocular injections for the anterior and posterior ocular diseases, respectively. Ophthalmic drug delivery, more than any other route of administration, may benefit to a full extent from the characteristics of nano-sized drug particles. In conclusion, a multi-disciplinary approach (from pharmacology to ophthalmology and from biomaterial science to pharmaceutical science) will bring, very soon, to a clinical use of these innovative nanosystems for the pharmacological management of sight-threatening eye diseases.
